# Long-Term Effects of Chronic Intermittent Ethanol Exposure in Adolescent and Adult Rats: Radial-Arm Maze Performance and Operant Food Reinforced Responding

**DOI:** 10.1371/journal.pone.0062940

**Published:** 2013-05-13

**Authors:** Mary-Louise Risher, Rebekah L. Fleming, Nathalie Boutros, Svetlana Semenova, Wilkie A. Wilson, Edward D. Levin, Athina Markou, H. Scott Swartzwelder, Shawn K. Acheson

**Affiliations:** 1 Department of Psychiatry and Behavioral Sciences, Duke University Medical Center, Durham, North Carolina, United States of America; 2 Neurobiology Research Laboratory, Durham Veterans Affairs Medical Center, Durham, North Carolina, United States of America; 3 Social Sciences Research Institute, Duke University, Durham, North Carolina, United States of America; 4 University of California San Diego, La Jolla, California, United States of America; Tulane University Medical School, United States of America

## Abstract

**Background:**

Adolescence is not only a critical period of late-stage neurological development in humans, but is also a period in which ethanol consumption is often at its highest. Given the prevalence of ethanol use during this vulnerable developmental period we assessed the long-term effects of chronic intermittent ethanol (CIE) exposure during adolescence, compared to adulthood, on performance in the radial-arm maze (RAM) and operant food-reinforced responding in male rats.

**Methodology/Principal Findings:**

Male Sprague Dawley rats were exposed to CIE (or saline) and then allowed to recover. Animals were then trained in either the RAM task or an operant task using fixed- and progressive- ratio schedules. After baseline testing was completed all animals received an acute ethanol challenge while blood ethanol levels (BECs) were monitored in a subset of animals. CIE exposure during adolescence, but not adulthood decreased the amount of time that animals spent in the open portions of the RAM arms (reminiscent of deficits in risk-reward integration) and rendered animals more susceptible to the acute effects of an ethanol challenge on working memory tasks. The operant food reinforced task showed that these effects were not due to altered food motivation or to differential sensitivity to the nonspecific performance-disrupting effects of ethanol. However, CIE pre-treated animals had lower BEC levels than controls during the acute ethanol challenges indicating persistent pharmacokinetic tolerance to ethanol after the CIE treatment. There was little evidence of enduring effects of CIE alone on traditional measures of spatial and working memory.

**Conclusions/Significance:**

These effects indicate that adolescence is a time of selective vulnerability to the long-term effects of repeated ethanol exposure on neurobehavioral function and acute ethanol sensitivity. The positive and negative findings reported here help to further define the nature and extent of the impairments observed after adolescent CIE and provide direction for future research.

## Introduction

Adolescence is a critical period for synaptic pruning and refinement across neocortical and non-neocortical regions (e.g., hippocampus). This process occurs throughout adolescence and continues into young adulthood [1]–[4]. Prolonged maturation in areas required for complex cognitive processes may be responsible for the limited planning, foresight and impulse control commonly observed among adolescents [5]. Deficits in these cognitive functions may also lead to increased novelty- and sensation-seeking [6]. Importantly, ethanol (EtOH) consumption is often initiated and occurs at its highest levels during adolescence and young adulthood [7]. These phenomena can coalesce, resulting in an escalation of risky behavior and the onset of alcohol use disorders (AUD) [8]–[10], which can adversely affect an individual's development into adulthood. As a result, a thorough understanding of the effects of ethanol during this developmental window is of great importance.

It is well established that acute EtOH consumption can have adverse effects on cognition and behavior in humans [11], [12]. Moreover, such effects vary with age. Children [13] and young adults [14] at one end of the developmental continuum and older adults [15] at the other end exhibit more sensitivity to the cognitive and behavioral disruption elicited by EtOH. This is corroborated by animal studies in which adolescent animals show less sensitivity to anxiogenesis [16]–[18]; the locomotor impairing effects [19], [20] and the hypnotic effects [21] of acute ethanol while being more susceptible to deficits in spatial memory acquisition [22].

There is also evidence that hippocampally mediated learning and memory may be developmentally sensitive to the effects of EtOH. For example, moderate acute EtOH exposure (1.0–2.0 g/kg) [22] but not higher (2.5 g/kg) or lower doses (0.5 g/kg) impairs acquisition of spatial memory more potently in adolescent rats than in adult rats [23]. Some electrophysiological evidence is consistent with these behavioral effects. Acute exposure of hippocampal slices to EtOH suppressed the induction of LTP and NMDA receptor-mediated synaptic activity, and enhanced extrasynaptic GABA_A_ receptor function more potently in slices from adolescent rats than in those from adults [24]–[27]. These findings notwithstanding, the neurodevelopmental sensitivity to ethanol on measures of learning and memory has not been observed consistently across species or memory tasks [28]–[31].

Given the prevalence of alcohol use during adolescence, and the differences in sensitivity to the acute effects of EtOH during this period, it is important to understand the long-term consequences of adolescent ethanol exposure. There is evidence that repeated EtOH exposure during adolescence can cause both neocortical and hippocampal damage [32], [33], and alter baseline GABA_A_ receptor-mediated function and its acute ethanol sensitivity in adulthood [34], [35]. One of the cognitive domains most vulnerable to disruption by both acute and chronic EtOH exposure is learning and memory. We have previously shown that repeated exposure to EtOH during adolescence promotes working memory deficits during EtOH challenge in adulthood when compared to age matched controls [36]. In the present study, we sought to expand that work by increasing the working memory delays from 1-hour to 3- and 6-hour delays and to assess the developmental trajectory of this adolescent window by including a young adult group.

We hypothesized that animals pre-treated with ethanol during adolescence would show greater signs of reference and working memory impairment than animals pre-treated as adults in the radial arm maze (RAM). We further hypothesized that late adolescent/young adult animals would be less impaired than the adolescents but more impaired than the adults in spatial working and reference memory tasks in the RAM. Therefore, the purpose of this study was to assess the enduring effects of chronic intermittent ethanol (CIE) exposure during adolescence, young adulthood and full adulthood on learning and memory in a hippocampally dependent task after all animals reached adulthood. Additional measures included behavioral intoxication scores and weight gain during CIE exposure, and assessment of BECs after acute ethanol challenges. In addition, an operant task assessing food-maintained responding under fixed and progressive ratio schedules of reinforcement was used to determine whether any observed deficiencies in reference or working memory in the RAM after CIE exposure and acute EtOH challenge were due to changes in food motivation or the reward value of food. Fixed ratio schedules, in which a fixed number of responses must be emitted for each food reward, assess the reward value of the food. Progressive ratio schedules, in which the the “cost” of the food reward is progressively increased over the duration of the session to determine the maximal effort the subject will exhibit to obtain the reward, provide a quantitative measure of incentive motivation for the food reinforcer [37]. In addition, the fixed and progressive ratio experiments allow us to evaluate the effects of CIE exposure on the learning of an operant task which does not involve either a working or a reference memory component. Thus, the results obtained from these fixed and progressive ratio schedule experiments facilitate the interpretation of any deficits in reference or working memory seen in the food-motivated RAM task after CIE exposure or EtOH challenges.

## Materials and Methods

All of the procedures used in this study were conducted in accordance with the guidelines of the American Association for the Accreditation of Laboratory Animal Care and the National Research Council's Guide for Care and Use of Laboratory Animals and were approved by the Durham VAMC, the Duke University and University of California San Diego Institutional Animal Care and Use Committees. The radial arm maze experiments were conducted at Duke University while the operant and blood ethanol level studies were conducted at the University of California San Diego.

### Animals and Chronic Intermittent Ethanol (CIE) Exposure

A total of 72 male Sprague-Dawley rats (n = 50 Duke site, n = 22 UCSD site; Charles River, USA) were double housed with *ad libitum* access to food and water. Animals were allowed to acclimatize for 9 days in the vivarium on a reverse 12∶12 hr light:dark cycle (lights off at 6am) prior to beginning CIE/saline (VWR, Suwanee, GA, USA) administration.

Animals were handled and treated with EtOH or saline as previously described in Fleming et al. [34]. Briefly, rats at PND30, PND50 and PND70 were used to represent adolescence, late adolescence/young adulthood, and adulthood, respectively, in the experiment assessing learning and memory (RAM), while additional groups of rats at PND30 and PND70 were used for the food-maintained operant tasks. All animals were exposed to a CIE exposure regimen consisting of 10 doses of 5 g/kg ethanol (35% v/v in saline at 18.12 mL/kg) or isovolumetric saline administered by intragastric gavage (IG) using a 2 days on, 2 days off intermittent schedule for 20 days followed by a 20 day washout period, thus allowing all animal to reach adulthood prior to behavioral testing.

### Radial-Arm Maze (RAM)

#### Apparatus and General Procedure

The 16-arm manual RAM apparatus was converted into a 12 arm maze by blocking four equidistant arms. These blocks remained in place throughout the habituation and testing phases and were distinguishable from the temporary blocks used during the delayed non-match to position task (DNMTP). Each arm was enclosed proximally (i.e., had walls 18 cm (H)×28 cm (L)) and the distal portions (28 cm) of the arms were open (i.e., had no walls). The maze arms (10 cm×60 cm) projected radially from the central area (50 cm in diameter). Animals began all trials in the holding area (27 cm in diameter) located in the central area. Various extra-maze cues were adhered to all walls within the testing room. All trials were recorded and analyzed using a USB video camera (LifeCam, Microsoft, Redmond, WA, USA) and ANY-Maze video tracking software (Stoelting, Wood Dale, IL, USA).

On day 7 of washout (7 days after the last CIE/saline administration) 18 adolescent, 16 young-adult and 16 adult rats were individually housed and began food restriction to 85% of normal weight in preparation for RAM. Animals remained on food restriction throughout training and had *ad libitum* access to water at all times except when undergoing behavioral testing. All food was given daily after completion of the task. Animals were handled again on washout days 14 through 17 prior to RAM apparatus habituation and training.

#### Habituation and Training Phase

Apparatus habituation was performed on day 18 of washout and consisted of one 15 m exploration with 1/3 Froot Loop® pieces placed throughout the maze, allowing the animals to become acquainted with the maze and the reward (this habituation phase and the following behavioral paradigms were modified from Terry et al. [38].

#### Win-Shift Phase (RAM)

Win-shift training was initiated 21 days after the last CIE exposure at a time when all animals had reached adulthood. Animals in the adolescent pre-treatment groups were at PND71, those in the young adult pre-treatment group were at PND91, and those in the adult pre-treatment group were at PND111. Once testing began, all animals were habituated to the RAM room for 30 m prior to each trial. Each animal was placed in the center circular ‘holding area’ and after a 30 s delay the animal was released and allowed to explore the maze. The same eight out of 12 arms were always baited, leaving the same four arms always un-baited. Each animal was given a pseudorandom un-baited arm combination that remained the same throughout all trials. Animals were removed from the apparatus after all eight food rewards were collected or after 15 m had elapsed, and were placed back in their home cage and returned to their housing room until their next trial on the following day. Animals were trained (1 trial per day) until they reached criterion for 3 consecutive days, or a maximum of 37 trials (if the criterion was never reached). The criterion was defined as≤2 type1 working memory errors &≤2 reference memory errors. A type1 working memory error was defined as a repeated entry into a given arm within a given trial. A reference memory error was defined as the first entry into any unbaited arm.Upon reaching criterion, or reaching the maximum number of trials, animals were switched to the DNMTP task.

#### Delayed Non-Match to Position (DNMTP) Phase (RAM)

Training consisted of two trials per day, with a 15 m delay between trials. Trial 1 was the information acquisition session, in which, four of the 12 arms were randomly selected and blocked while all available arms were baited. Each animal was placed back in its home cage after all 8 rewards were retrieved or after 10 m had elapsed. After a 15 m delay the animals were placed back in the maze for Trial 2 with all 12 arms available for exploration. The test session was terminated when the animal retrieved all four rewards from the previously blocked arms. Each day a different set of 4 arms were pseudorandomly blocked. Once criterion (≤1 type1 working memory error or type2 working memory error - entry into an unbaited arm, in trial 2, for 3 consecutive days.), or the maximum number of training days (17) was reached (see Table 1 for further parameter details), animals were tested with longer delays (1 hr, 3 hr and 6 hrs), each of which was presented twice in pseudo-random order (n = 1 adolescent saline, n = 0 adolescent CIE, n = 1 young adult saline, n = 2 young adult CIE, n = 2 adult saline, n = 0 adult CIE failed to reach criterion at the 15minute delay).

**Table 1:** Description of trials to criterion and the dependent measures used in the RAM task.

Parameter Name	RAM Phase	Description
Trials to criterion	Win-Shift	≤2 type 1 working memory errors &≤2 reference memory errors, for 3 consecutive days
Reference memory error	Win-Shift	1^st^ entry into all unbaited arms
Trials to criterion	DNMTP	≤1 total error in trial 2, for 3 consecutive days
Working memory error - Type1	Win-Shift & DNMTP	Repeated entries into the same arm within the same trial
Working memory error - Type2	DNMTP	Entries into unbaited arms in trial 2
Speed	Win-Shift & DNMTP	Time active/distance traveled (m/s)
Trial duration	Win-Shift & DNMTP	Time to complete the trial (s)
Distance traveled	Win-Shift & DNMTP	Distance traveled (m)
% Time in open arm	Win-Shift & DNMTP	(Total time spent in open arms/trial duration) x 100

#### Acute EtOH Challenge during DNMTP Phase (RAM)

Immediately following completion of the psuedorandomized delays, animals were tested for one additional day on the DNMTP task, with an acute EtOH challenge. The acute EtOH challenge was administered, on average, 67 days (±1.17 s.e.m.) after the last CIE dose. In this phase of testing, all animals, regardless of age or pre-treatment condition, were given one acute 1.5 g/kg EtOH challenge by intraperitoneal injection (i.p.) 30 m prior to the acquisition trial (Trial 1). The purpose of this challenge was to determine whether earlier CIE exposure resulted in changes in sensitivity to acute EtOH. Maze performance was assessed during acquisition (Trial 1: 30 m post-injection), and following a 1-hour delay (Trial 2: 90 m post-EtOH challenge). Duration, distance traveled, speed and working memory errors were assessed during the acquisition phase and all previously described dependent measures (see Table 1) were assessed in the information recall session (Trial 2).

### Acquisition and Maintenance of Food Fesponding under Fixed and Progressive Ratio Schedules of Reinforcement

#### Apparatus and General Procedure

Behavioral testing was conducted in Plexiglas operant chambers (24 cm×30 cm×28 cm; Med Associates, St. Albans, VT, USA) fully enclosed within sound and light attenuating boxes. One wall of the chamber was equipped with two levers (3 cm×1.8 cm each, 3 cm above the floor), only one of which was active. Responses on the active lever resulted in delivery of a single 45 mg food pellet reward (TestDiet, Richmond, IN, USA) as determined by the reinforcement schedule. Responses to the inactive lever were recorded but had no scheduled consequences. A food receptacle was located between the two levers and a house light was located on the opposite wall. All programs and data collection was controlled by a computer running MED-PC IV software (Med Associates, St. Albans, VT, USA).

Both CIE-exposed (n = 6 adolescent; n = 5 adult) and saline-exposed (n = 6 adolescent; n = 5 adult) rats used to assess food-motivated responding were weighed before the first IG gavage of each binge cycle. CIE-exposed animals were observed for behavioral signs of intoxication [39], [40] after EtOH IG gavage on days 1, 2, and 3 of CIE exposure. Briefly, rats were observed for behavioral indicators of CNS depression 45–75 m after each injection and assigned a behavioral intoxication score from 0 to 5. Rats used in this experiment remained pair-housed throughout the study. Food-restriction (18 g of food per day per rat in addition to food pellets earned in the operant conditioning boxes) began on day 20 of the EtOH/saline-free washout and continued for the duration of the study. FR training was initiated on day 22 of the washout when all animals had reached adulthood (adolescent exposure group PND70; adult exposure group PND110). All rats had *ad libitum* access to water at all times except when undergoing behavioral testing.

#### Acquisition and Maintenance of Food Responding under Fixed and Progressive Ratio Schedules of Reinforcement

Rats were first exposed to a fixed ratio (FR1), timeout 1 s (FR1 TO1 s) schedule of reinforcement wherein each lever press response produced a food pellet reward followed by a 1 s timeout. Responses emitted during the timeout were recorded but never produced food pellet rewards. Training schedules of reinforcement progressed from FR1 TO1s to FR2 TO10s, FR5 TO20s and finally FR10 TO20s. Criterion for advancement to the next training schedule was 100 reinforced responses within a 60 m session. In the FR10 TO20s schedule, each session was always 60 m in duration. Rats were tested on FR10 TO20s schedule of reinforcement until stable performance was achieved (<10% variability in response rate across five consecutive sessions).

After all rats completed training and testing under the FR schedule of reinforcement, the progressive ratio (PR) schedule was initiated. In this schedule, the “cost” of the reward (e.g., food pellet) was progressively increased over the duration of the session to determine the maximal effort the subject will exhibit to obtain the reward. The response requirement progression was according to the formula {5e^[(pellet#+2)/4]^}−6. That is, the progression of required lever-presses to earn one pellet was 5, 8, 11, 16, 23, 31, 41, 55, 72, 94, 123, 160, 207, 267, 345, etc. The session was terminated if no reinforcer was earned for one hour or 6 h had elapsed. The last ratio value successfully completed is defined as the breaking point, and is a measure of incentive-motivation [37], [41].

#### Responding under Fixed-Ratio Reinforcement Schedule after Acute EtOH Challenges

After completion of training under the PR schedule of reinforcement, stable responding in the FR schedule was re-established. Rats were then administered three doses of EtOH (1, 2, 3.5 g/kg) and vehicle via gavage according to a within-subjects Latin square experimental design with at least four days of testing under baseline conditions between each acute EtOH challenge. On days when an acute EtOH challenge was administered, tail-tip blood samples were collected for analysis of blood EtOH concentrations (Analox Instruments, Ltd., Lunenberg, MA, USA) approximately 70–85 m post-gavage, depending on the time taken by the rats to complete the test session.

### Statistical Analysis

One animal was removed from the RAM study due to failure to complete any trials during the win-shift phase. In addition, all data from animals that failed to reach criterion on the training phase of DNMTP were removed from all DNMTP analyses. All analyses were performed using SPSS 18 (UCSD site) or 19 (Duke site; SPSS Inc., Chicago, IL). Statistical significance was assessed using an alpha level of 0.05. For RAM data, 2-way (*Age*×*Pre-treatment*) analyses of variance (ANOVA) were conducted on all non-repeated dependent measures (e.g., trials to criterion). When assessing the effect of *Age* and *Pre-treatment* over time (e.g., across days or trials), a 3-way (*Age*×*Pre-treatment*×*Day/Trial*) repeated measures ANOVA (RM-ANOVA) was employed. Where p<0.05, appropriate t-tests or one-way ANOVAs were performed to test simple main effects. Planned pairwise comparisons were performed in the presence of ordinal interactions [42].

Where our primary hypotheses (i.e., *Age*×*Pre-treatment* interactions) were non-significant, we reported effect size in the form of partial eta squared (η_p_^2^) and the corresponding 90% confidence interval. The η_p_^2^ effect size represents the proportion of variance in a particular dependent variable (e.g., working memory errors) that is accounted for by the selected effect (e.g., *Age*×*Pre-treatment* interaction), controlling for all other effects in the model. As a result, η_p_^2^ is bound by 0 and 1.0. That is, an independent variable cannot account for less than 0.0% or more than 100% of the variance in a dependent variable. Confidence intervals for η_p_^2^ were calculated according to methods described by [43] using the corresponding SPSS syntax code [44]. These effect sizes are discussed relative to a criterion effect of 0.05. Estimates of effect size (η_p_^2^+90% confidence interval) falling below this criterion are described as functionally irrelevant. That is, if we are 90% confident that the *Age*×*Pre-treatment* interaction accounts for less than 5% of the variance in a particular dependent variable, then that effect is to be considered functionally irrelevant. Conversely, estimates of effect size (η_p_^2^+90% confidence interval) that overlap or exceed this 5% criterion, but otherwise remain statistically non-significant, are to be considered of some potential functional relevance and may be worthy of further investigation. This approach is an extension of the equivalence testing approach described elsewhere using alternative effect size measures [e.g., [45]–[48]]. Further details of this approach are presented in the Discussion below.

For the fixed and progressive ratio experiments, data were analyzed using repeated measures ANOVA. The Mauchly's test of sphericity of the covariance matrix was applied. When the sphericity assumption was violated, the degrees of freedom for any term involving that factor were adjusted to more conservative values by applying the Huynh-Feldt correction [49]. Corrected degrees of freedom were reported to 1 decimal place. Post-hoc comparisons among individual means were made using “simple effects” ANOVAs and t-tests with a Šidák adjustment for multiple comparisons. When comparisons were made amongst factors with three levels, no correction for multiple comparisons were required (Fisher's Least Significant Difference procedure).

## Results

### Assessment of Learning and Memory in the Radial Arm Maze (RAM)

#### Win-shift Phase

**Figure 1:**
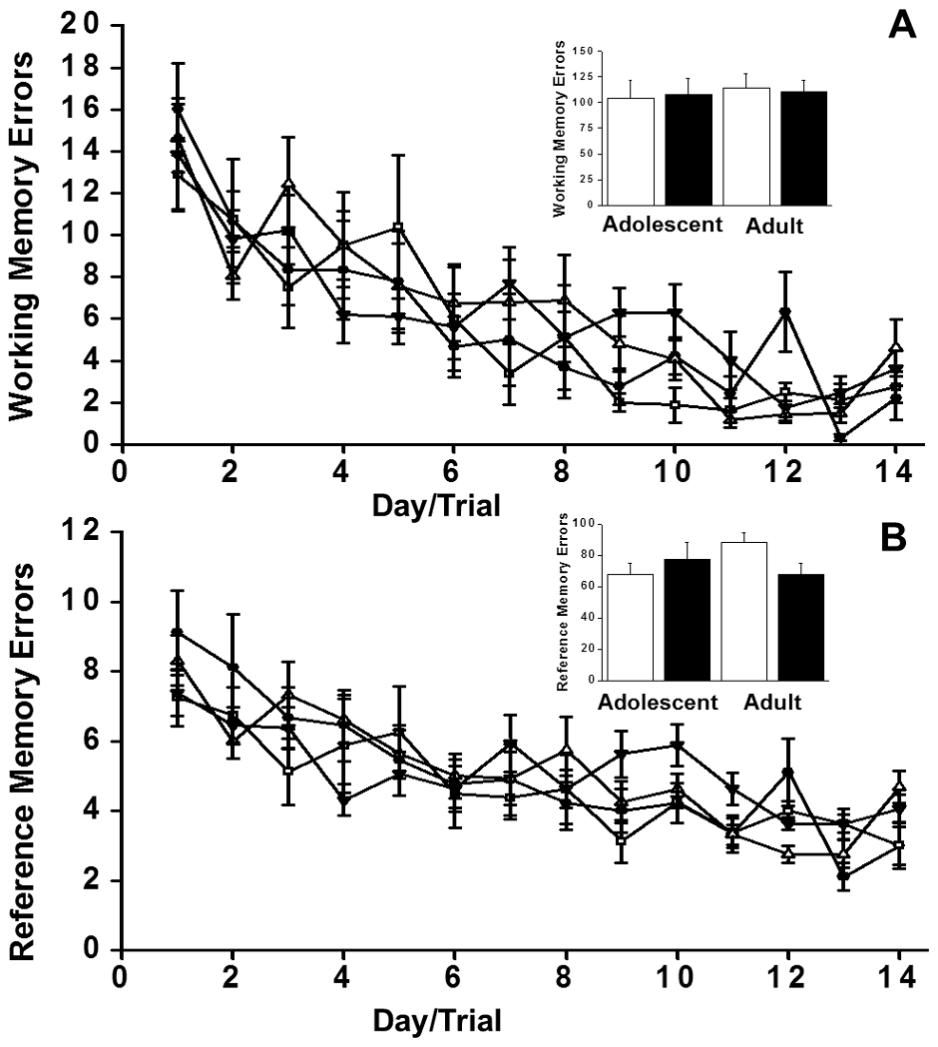
Effects of chronic intermittent ethanol (• and ▾) or saline (□ and ▵) exposure during adolescence or adulthood (respectively), on working memory (panel A) and reference memory (panel B) over the 1st 14 days of training in the initial ‘Win-Shift’ phase of RAM testing. The number of working memory (p<0.05) and reference memory (p<0.05) errors decreased across days. The effect of Age and Pre-Treatment were not significant. The histogram insets illustrate the overall effect (i.e., cumulative over all sessions) of ethanol (black bars) and saline (white bars) on working (panel C) and reference (panel D) memory errors. Data are expressed as means ±SEM (n = 9 adolescent saline, n = 9 adolescent CIE, n = 8 young-adult saline, n = 8 young-adult CIE, n = 8 adult saline, n = 8 adult CIE).

There were no *Age*×*Pre-treatment* interactions and no main effects of *Age* involving young adult animals that differentiated them from adult animals. Therefore, the young adult and adult groups were combined for all subsequent analyses. During the Win-shift phase, data were analyzed across the first 14 days of learning (the point at which the first animal reached criterion) to investigate differential effects on learning curves. Learning was assessed by the decline in the number of working and reference memory errors (Figure 1A and 1B, respectively). Because animals were trained to criterion, data were also analyzed using cumulative errors to criterion as the dependent measure (see insets in Figure 1A and 1B). Therefore, only animals that reached criterion were used in these analyses. There was no substantive difference in the frequency with which animals failed to reach criterion between the various Age x Pre-treatment groups (3 adolescent saline, 3 adolescent CIE, 3 young-adult saline, 1 young adult CIE, 2 adult saline, and 2 adult CIE failed to reach criterion).

Both working (*F*_13,585_ = 19.55, *p*<0.001), and reference (*F*_13,585_ = 15.70, *p*<0.001) memory errors decreased across the first 14 days of training, indicative of learning across this initial training phase (Figure 1A and 1B, respectively). However, there were no main effects of *Age or Pre-treatment, and no Age*×*Pre-treatment* interactions on any of the additional parameters measured, including: mean speed; distance traveled; duration and trials required to reach criterion (all *p*'s≥0.11; Figure 1). The interaction between *Age*, *Pre-treatment*, and learning across the 14 days (*Age*×*Pre-treatment*×*Day*) accounted for only 2.1% of the variance in working memory (η_p_^2^ = 0.021) and reference memory errors (η_p_^2^ = 0.021). Collapsing across these learning days, the interaction between *Age* and *Pre-treatment* (*Age*×*Pre-treatment*) accounted for 0.1% of the variance in cumulative working memory (η_p_^2^ = 0.001) and 3% of the variance in cumulative reference memory (η_p_^2^ = 0.03) errors.

#### DNMTP Training Phase (15 minute delay)

The transition from Win-shift to DNMTP requires that animals learn new rules while retaining some rules from the previous phase. We therefore assessed whether there was an *Age* or *Pre-treatment* dependent deficit in “reversal-like” effects on day 1 of the DNMTP. The number of type1 memory errors (repeated entries into the same arm within the same trial) did not differ significantly based on *Pre-treatment*, *Age*, or due to an *Age*×*Pre-treatment* interaction (all *p*'s>0.49). In addition, there were no *Age*, *Pre-treatment* or *Age*×*Pre-treatment* interactions in type2 memory errors (entries into unbaited arms in trial 2/previously baited in trial 1) (all p's>0.38). The *Age*×*Pre-treatment* interaction accounted for<1% of the variance in type1 memory errors (η_p_^2^ = 0.009) and<2% of the variance in type2 memory errors (η_p_^2^ = 0.017). These data suggest that there were no effects of adolescent or adult CIE on “reversal-like” measures during the transition from Win-Shift to DNMTP.

**Figure 2:**
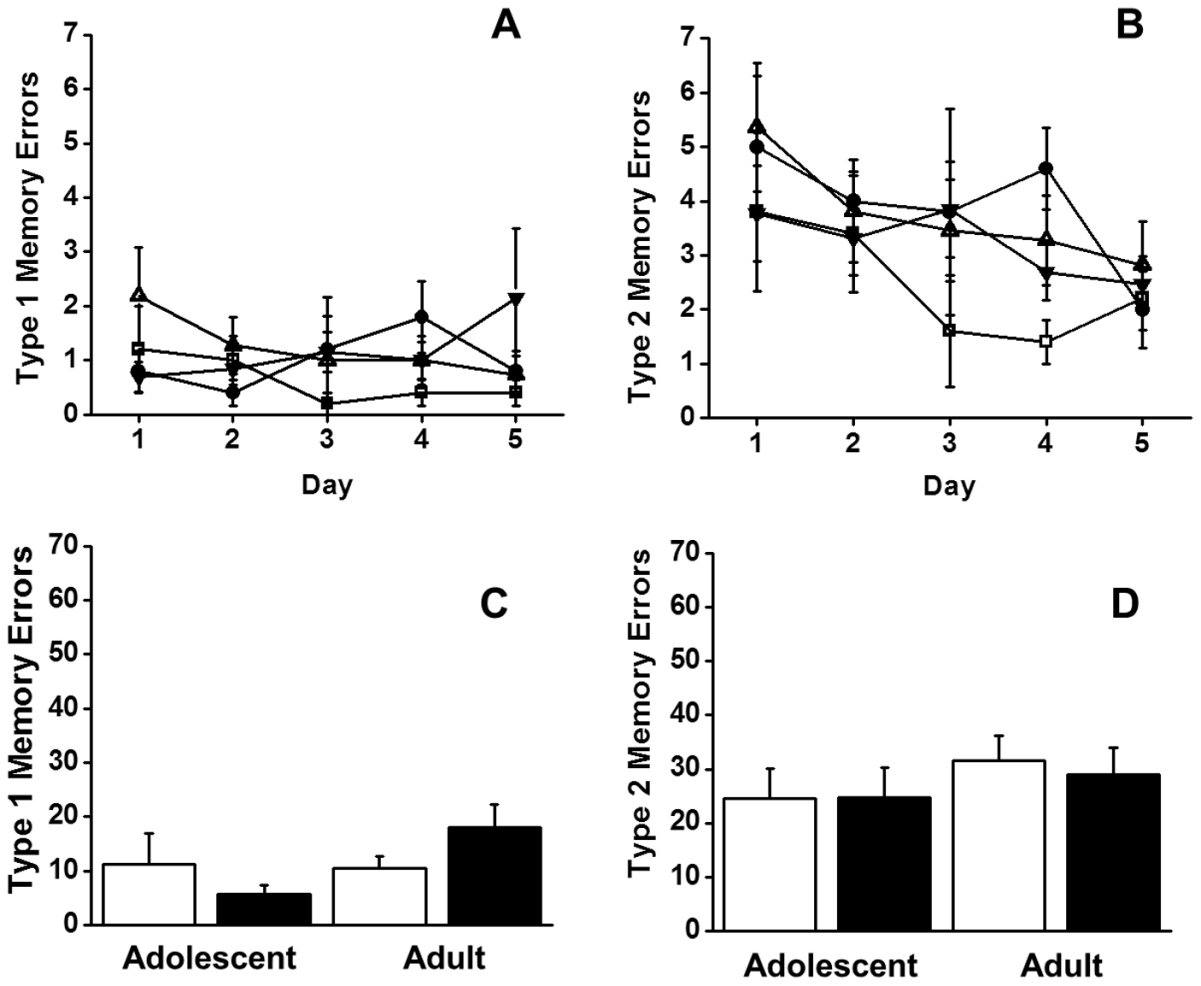
Effects of chronic intermittent ethanol (• and ▾) or saline (□ and ▵) exposure during adolescence or adulthood (respectively), on type1 working memory (panel A) and type2 working memory (panel B) errors over the 1st 5 days of training in the DNMTP. The histograms represent the cumulative effect of ethanol (black bars) and saline (white bars) on type1 (panel C) and type2 (panel D) working memory errors. Data are expressed as means ±SEM (n = 5 adolescent saline, n = 5 adolescent CIE, n = 5 young-adult saline, n = 7 young adult CIE, n = 6 adult saline, n = 6 adult CIE).

Type1 and type2 errors were also assessed over the first 5 days (the time at which the first animals reached criterion) allowing us to look more closely at the initial learning phase. There was no significant effect of *Day* on type1 memory errors (*p* = 0.955, Figure 2A) indicating that the rule regarding arm re-entries, previously learned in the Win-shift phase, transferred to the DNMTP phase. In addition, there was no interaction of *Day*×*Age*×*Pre-treatment* and no main effects of *Age*, *Pre-treatment*, or *Age*×*Pre-treatment* interactions on type1 memory errors (all *p*'s>0.40). The number of type2 memory errors (entering an arm that was previously baited in trial 1) decreased across training as expected (*F_4,120_* = 17.48, *p* = 0.02, Figure 2B), indicating that the animals were able to learn the new rules in order to complete the task. However, there was no *Day*×*Age*×*Pre-treatment* interaction and no main effects of *Age*, *Pre-treatment*, or *Age*×*Pre-treatment* interactions on type2 memory errors (all *p*'s>0.141). The interaction between *Age*, *Pre-treatment*, and learning across the first 5 days (*Age*×*Pre-treatment*×*Day*) accounted for 1.8% and 2% of the variance in type1 memory errors (η_p_^2^ = 0.018) and type2 memory errors (η_p_^2^ = 0.020) respectively. Collapsing across training days, the interaction between *Age* and *Pre-treatment* (*Age*×*Pre-treatment*) accounted for 0.7% of the variance in cumulative type1 memory errors (η_p_^2^ = 0.007) and 7% of the variance in cumulative type2 memory (η_p_^2^ = 0.071) errors.

The numbers of trials required to reach criterion did not differ significantly based on *Pre-treatment, Age* or on the interaction of *Pre-treatment*×*Age* (adolescent saline = 4; adolescent ethanol = 5; adult saline = 5; adult ethanol = 3, all *p*'*s*≥0.20). There were no main effects (*Age* or *Pre-treatment*) and no interaction on cumulative number of type1 and type2 working memory errors (see Figure 2C and 2D), mean trial duration, speed or distance traveled when collapsed across training days (all *p*'s≥0.09). The *Age*×*Pre-treatment* interaction accounted for∼1% of variance in the number of trials required to reach criterion (η_p_^2^ = 0.013); 7% and 0.6% of variance in the cumulative number of type1 and type2 working memory errors respectively (η_p_^2^ = 0.07, η_p_^2^ = 0.006); and, 0% of variance in mean trial duration (η_p_^2^ = 0.000).

**Figure 3:**
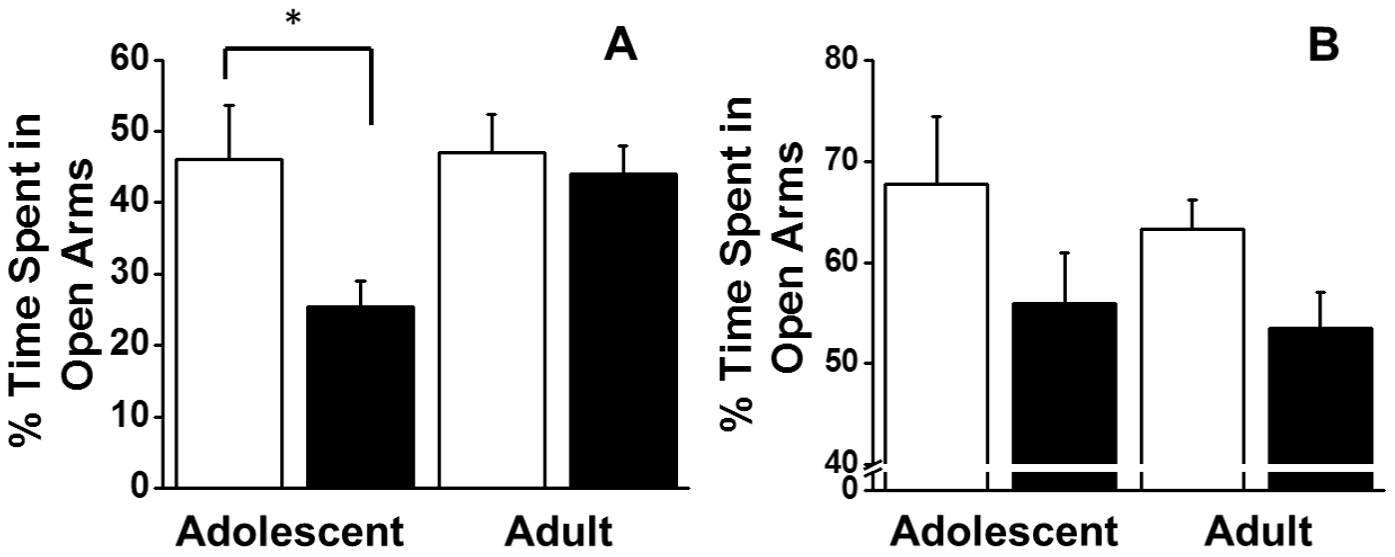
Effects of chronic intermittent ethanol (black bars) or saline (white bars) exposure during adolescence or adulthood after the 15 m delay, (during training, panel A) and after the 3 h delay (panel B) on%time spent in the open arms. Data is expressed as group mean ± SEM (n = 5 adolescent saline, n = 5 adolescent CIE, n = 5 young adult saline, n = 7 young-adult CIE, n = 6 adult saline, n = 6 adult CIE). Asterisk denotes a significant difference between the animals pre-treated in adolescence with ethanol vs. saline (*Post-hoc* t-test, *p*<0.05).

Curiously, during the course of data collection, some animals were observed to spend relatively little time in the distal most portions of the RAM arms, which were not enclosed by walls. We pursued this observation by analyzing the percent time animals spent in the open portions of the arms. There was a clear effect of *Pre-treatment* on the percentage of time spent in the open portions of the maze arms (*F_1,30_* = 4.318, *p* = 0.046). However, there was no *Age* effect (*F_1,30_* = 2.965, *p* = 0.095) or *Age*×*Pre-treatment* interaction (*F_1,30_* = 2.3825, *p* = 0.133) (Figure 3A). Visual inspection of Figure 3A revealed an ordinal interaction, and further analysis indicated that animals exposed to CIE during adolescence spent less time in the open portions of the maze arms than did their age-matched controls (*t_11_* = 1.805, *p* = 0.04), whereas the adults with the same history of CIE exposure did not show this effect. No such effects were observed in the Win-shift phase of the RAM experiment.

#### DNMTP Random Delays

Under 1-hour delay conditions, there were no main effects (*Pre-treatment* or *Age*), or interactions on working memory errors (type1 or type2), locomotor measures (speed and distance traveled), trial duration or time spent in the open portion of the arms (all *p*'s≥0.10). During the 1-hour delay, the *Age*×*Pre-treatment* interaction accounted for 1% of the variance in type1 working memory errors (η_p_^2^ = 0.01) and<1% of the variance in type2 working memory errors (η_p_^2^ = 0.003).

Under the 3-hour delay conditions, there were no main effects (*Pre-treatment* or *Age*) or interactions on working memory errors (type1 or type2), speed, distance traveled or trial duration (all *p*'s≥0.10). The *Age*×*Pre-treatment* interaction accounted for approximately 1% or less of the variance in type1 (η_p_^2^ = 0.013) and type2 (η_p_^2^ = 0.004) working memory errors. However, there was a main effect of *Pre-treatment* on time in the open arms, with CIE animals spending less time in the open portion of the arms than control animals (*F_1,30_* = 5.55, *p* = 0.03; Figure 3B).

**Figure 4:**
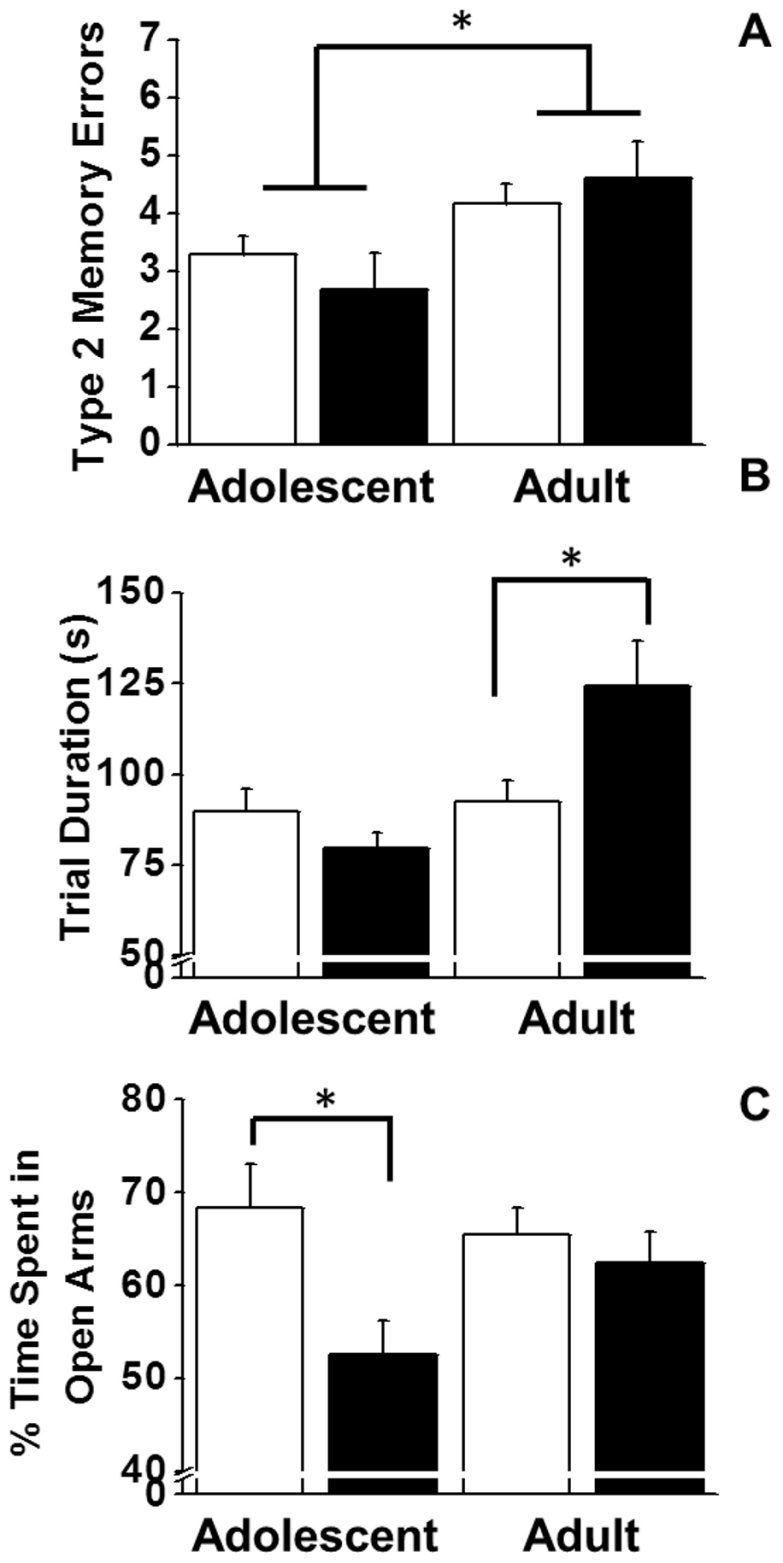
Effects of chronic intermittent ethanol (black bars) or saline (white bars) exposure during adolescence or adulthood after the 6 h delay in DNMTP. Data on type2 working memory errors (panel, A), trial duration (panel, B) and%time spent in the open arms (panel C) are expressed as group mean ± SEM (n = 5 adolescent saline, n = 5 adolescent CIE, n = 5 young adult saline, n = 7 young adult CIE, n = 6 adult saline, n = 6 adult CIE). Asterisk denotes a significant age difference when collapsed over treatment (panel A) and a significant difference between animals treated in adulthood with ethanol vs. saline (panel B). (*Post-hoc* t-test, *p*<0.05).

Following the 6-hour delay, there were no main effects (*Pre-treatment* or *Age*) or interactions on type1 working memory errors (*p*'s>0.13). However, this prolonged delay did reveal a clear effect of *Age* on type2 working memory errors, with older animals making more errors than the younger animals (*F_1,30_* = 4.150, *p* = 0.05; Figure 4A). When locomotor measures (i.e., speed and distance) were assessed there was no effect of *Age* or *Pre-treatment* (all *p*'s≥0.12). However, there was a nearly significant *Age* effect on the time required to complete the trials, i.e., trial duration. Specifically, older animals required more time to complete trials than did the younger animals (*F_1,30_* = 4.04, *p* = 0.052; Figure 4B). Visual inspection of Figure 4B revealed an ordinal interaction and further analysis indicated that animals pre-treated with CIE as adults required more time to complete the trials than did their aged-matched controls (t_22_ = 2.30, *p* = 0.02; Figure 4B). There was also a significant main effect of *Pre-treatment* on time in the open arms during the 6-hour delay (*F_1,30_* = 5.60, *p* = 0.03; Figure 4C). Moreover, visual inspection of Figure 4C also revealed an ordinal interaction indicating that animals exposed to CIE in adolescence spent significantly less time in the open arms compared to age-matched controls (*t_8_* = 2.67, *p* = 0.01). No such effect was observed in the animals treated in adulthood.

#### DNMTP Acute Ethanol Challenge (one hour delay)

**Figure 5:**
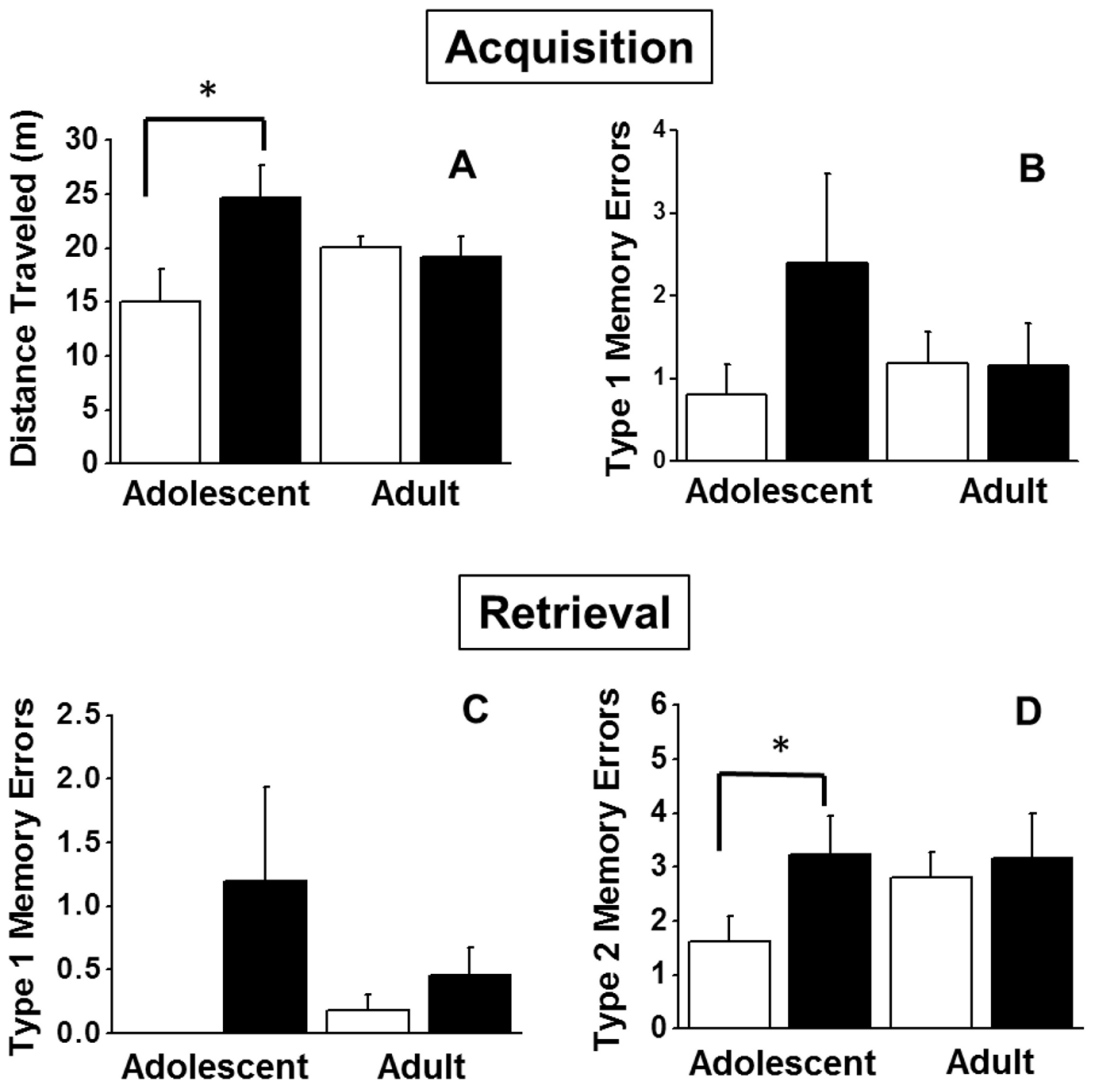
Effect of acute ethanol following chronic intermittent ethanol (black bars) or saline (white bars) exposure during adolescence or adulthood. Panel A and B represent distance traveled and type1 working memory errors, respectively, 30 m after administration of 1.5 g/kg EtOH i.p. during the acquisition phase of DNMTP. Bottom panels C and D represent type1 and type2 working memory errors, respectively, 90 m after administration of 1.5 g/kg EtOH i.p. during the retrieval phase of DNMTP. Data are expressed as group means + SEM (n = 5 adolescent saline, n = 5 adolescent CIE, n = 5 young adult saline, n = 7 young adult CIE, n = 6 adult saline, n = 6 adult CIE). Asterisks denote a significant difference between the animals pre-treated with ethanol vs. saline during adolescence (All *Post-hoc* t-tests, *p*<0.05).

There was a significant *Age*×*Pre-treatment* interaction on distance travelled during the information acquisition session (*F_1,30_* = 3.67, *p* = 0.03; Figure 5A). Tests of simple main effects revealed a significant increase in the distance traveled (*t_8_* = 2.24, *p* = 0.03) in animals exposed to CIE during adolescence compared to age matched controls. There was no differential effect of acute EtOH on type1 working memory errors during the acquisition trial (Figure 5B). However, after the 1-hour delay (90 m after the EtOH challenge dose) animals exposed to CIE made significantly more type1 working memory errors than animals exposed to saline (*F_1,30_* = 5.88, *p* = 0.02; Figure 5C), independent of the animals' age at the time of exposure. The main effect of *Pre-treatment* on type 2 working memory errors approached significance (*F_1,30_* = 3.27, *p* = 0.08; Figure 5D). Visual inspection of figure 5D also reveals an ordinal interaction and follow-up tests of simple main effects reveal that animals exposed to CIE in adolescents made significantly more type2 working memory errors than age matched controls after acute EtOH challenge (t_22_ = 2.34, *p* = 0.02).

### Fixed and Progressive Ratio Food Responding

#### Behavioral Intoxication Scores and Weight Gain in Rats used to Assess Food-Motivated Responding

**Figure 6:**
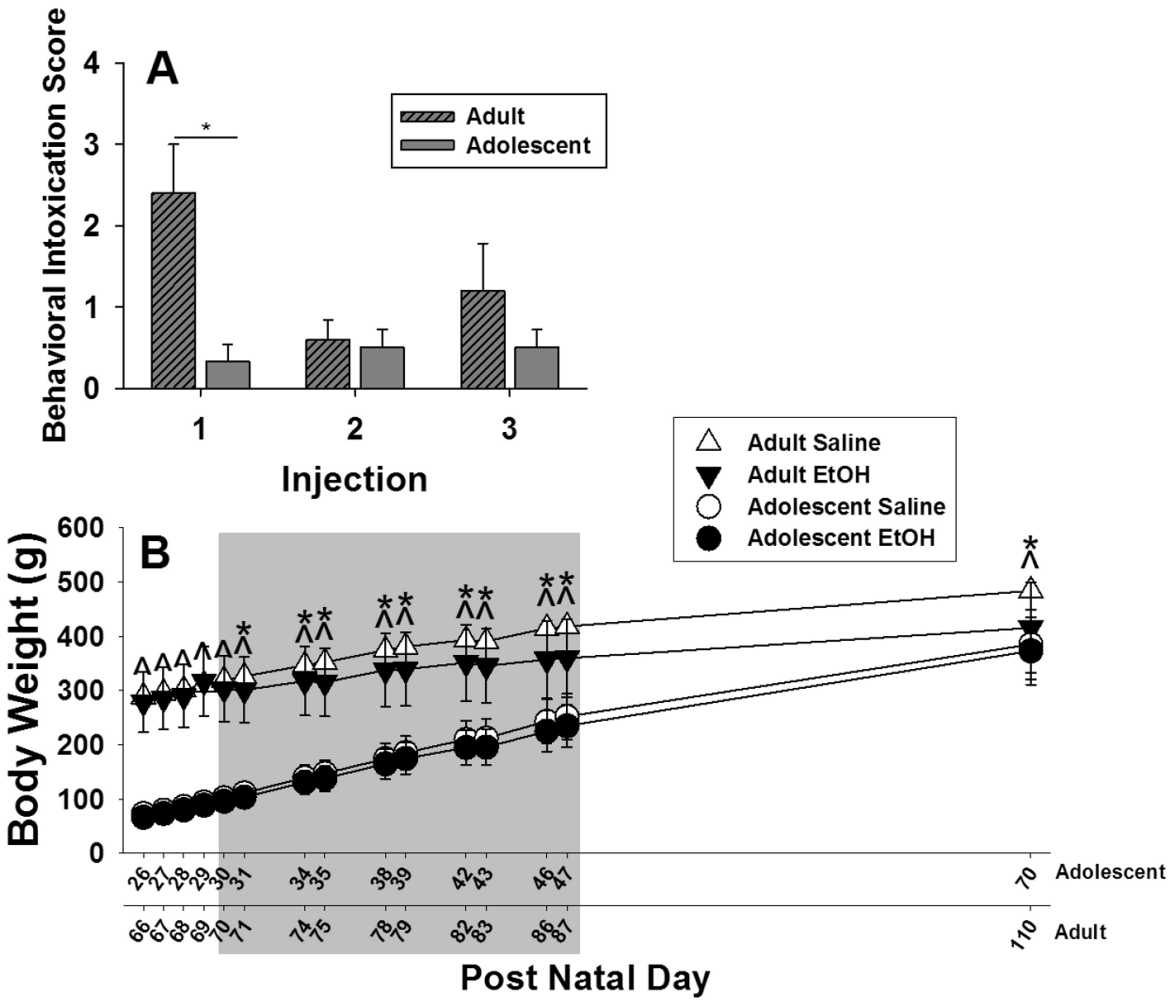
Behavioral intoxication scores after the first three CIE EtOH administrations (panel A) and body weights before, during and after CIE (panel B). Data are expressed as group mean ± SEM. For the behavioral intoxication score, the asterisk denotes a significant effect of age (adolescent vs. adult independent of CIE exposure) on a specific day (*Post-hoc t*-test with a Šidák adjustment for multiple comparisons, *p*<0.05). For the body weights, carats denote a significant effect of age at treatment (adult-treated vs. adolescent-treated independent of CIE exposure). Asterisks denote a significant effect of CIE exposure (ethanol vs. saline) in only adult CIE-exposed rats on a specific day (*Post-hoc t*-test with a Šidák adjustment for multiple comparisons, *p*<0.05). There were no significant differences in body weights between CIE- and saline-exposed adolescent rats.

During CIE exposure, behavioral intoxication was evaluated after ethanol doses 1, 2 and 3 (Adolescent group PND30, 31 and 34; Adult group PND70, 71 and 74; Figure 6A). There was a significant *Administration Number*×*Age* interaction (*F_2,18_* = 5.66, *p*<0.05) that *post-hoc* comparisons attributed to behavioral intoxication scores being significantly higher in adult rats compared to adolescent rats after the first EtOH administration (PND30 and 70 in adolescent and adult rats, respectively), with no significant group differences after the second or third EtOH exposure.

Rats were weighed for four days before the CIE exposure period, immediately before each ethanol or saline injection during the CIE exposure period, and then 23 days after the final injection (Figure 6B). There was a significant three-way *Day*×*Age*×*Pre-treatment* interaction (*F_14,352_* = 2.65, *p*<0.005). *Post-hoc* tests revealed that saline-exposed adult rats had higher body weights than their CIE-exposed age-matched counterparts throughout the CIE period. There were no differences between CIE-exposed and saline-exposed adolescent rats.

#### Blood Ethanol Concentrations (BECs) after Acute Ethanol Challenges (Fixed Ratio Responding Task)

During the acute EtOH challenges, ANOVA on the BECs revealed a significant main effect of *Dose* (*F_2,34_* = 11.93, *p*<0.001), but no effect of *Pre-treatment*, *Age* or interaction effects. *Post-hoc* tests indicated that BECs were higher when EtOH was administered at the doses of 2 g/kg (adolescent-CIE: 47.45±8.02 mg/dl; adolescent-saline: 92.58±23.60 mg/dl; adult-CIE: 66.52±13.17 mg/dl; adult saline: 84.60±20.79 mg/dl) and 3.5 g/kg (adolescent-CIE: 68.03±9.05 mg/dl; adolescent-saline: 100.78±13.89 mg/dl; adult-CIE: 87.60±18.57 mg/dl; adult saline: 120.10±27.85 mg/dl) compared to EtOH dose of 1 g/kg (adolescent-CIE: 21.12±6.05 mg/dl; adolescent-saline: 60.28±21.01 mg/dl; adult-CIE: 50.54±12.27 mg/dl; adult saline: 33.08±11.43 mg/dl).

#### Acquisition and Maintenance of Food Responding under Fixed and Progressive Ratio Schedules of Reinforcement

**Figure 7:**
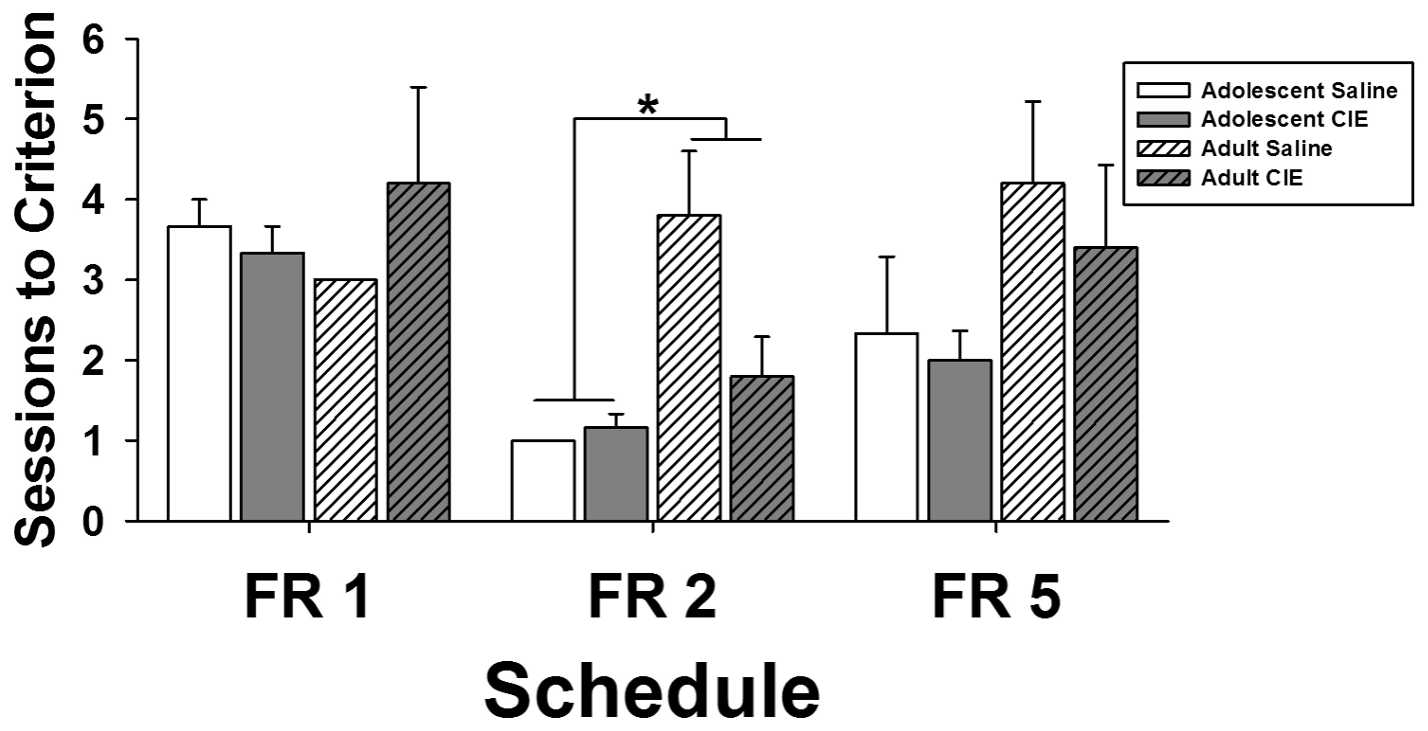
Effects of chronic intermittent ethanol or saline exposure during adolescence or adulthood on number of training sessions required to meet criteria for advancement in each of the three Fixed Ratio (FR) training schedules. Data are expressed as group mean + SEM (n = 6 adolescent-saline, n = 6 adolescent-CIE, n = 5 adult-saline, n = 5 adult-CIE). Asterisk denotes a difference in the number of training sessions required to reach criterion for rats exposed to CIE/saline as adolescents compared to rats exposed to CIE/saline as adults independent of CIE exposure (simple effects ANOVA, *p*<0.05).

Exposure to CIE or saline had no effect on acquisition of lever-press responding on FR schedules of reinforcement with increasing response requirements. There were significant main effects of S*chedule* (*F_2,36_* = 6.56, *p*<0.001) and A*ge* (*F_1,18_* = 8.19, *p*<0.05) on the number of sessions required to reach criterion (Figure 7). *Post-hoc* tests showed that adolescent exposed rats required fewer sessions than adult exposed rats to reach criterion on the FR 2 schedule. There was no significant main effect of CIE exposure and no significant interactions.

All rats were tested on the FR10 TO20s (FR10 timeout 20 seconds) schedule of reinforcement for 5 days followed by 5 days of testing under the PR schedule of reinforcement. ANOVAs revealed no effect of *Pre-treatment*, *Age* or their interaction on responding under either FR or PR schedules of reinforcement (Table 2).

**Table 2:** Response rate (responses/min) and Pellets earned per session in the final 5 sessions of both the Fixed Ratio 10 TO 20 s and Progressive Ratio experiments.

Schedule	Treatment group	Response Rate	Pellets Earned Per Session
Fixed Ratio	Adolescent CIE-exposed	69.20±15.39	23.00±4.80
	Adolescent Saline-exposed	53.49±9.66	23.99±8.31
	Adult CIE-exposed	60.77±19.03	16.43±2.71
	Adult Saline-exposed	47.58±10.11	16.77±4.21
Progressive Ratio	Adolescent CIE-exposed	110.67±16.64	13.53±0.90
	Adolescent Saline-exposed	106.13±11.40	13.33±1.05
	Adult CIE-exposed	110.56±8.90	12.44±0.61
	Adult Saline-exposed	103.32±9.00	12.40±0.85

Data are expressed as group mean ±SEM. There were no significant differences attributable to either *Age* at treatment (adolescent vs. adult) or *Treatment* (CIE or saline).

#### Responding under Fixed-Ratio Reinforcement Schedule after Acute Ethanol Challenges

**Figure 8:**
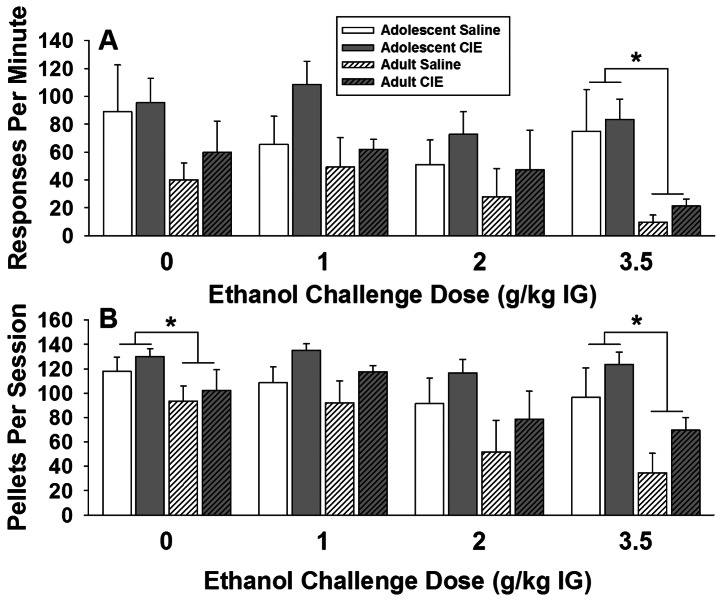
Effects of chronic intermittent ethanol or saline exposure during adolescence or adulthood on response rate (top panel, A) and average pellets per session (bottom panel, B) after an acute ethanol challenge. Data are expressed as group mean + SEM (n = 6 adolescent-saline, n = 6 adolescent-CIE, n = 5 adult-saline, n = 5 adult-CIE). Asterisks denote a significant difference between rats exposed to CIE or saline as adults versus adolescents during acute ethanol challenge at specific doses (simple effects ANOVA, *p*<0.05).

There was no significant effect of *CIE Exposure* on either response rate, or the total number of pellets earned amongst rats exposed to CIE during adolescence or adulthood. Nevertheless, there was a significant main effect of *Ethanol Dose* on response rate (*F_3,54_* = 4.51, *p*<0.01; Figure 8A) and *Number of Pellets Earned* (*F_3,54_* = 7.88, *p*<0.001, Figure 8B). Independent of age and CIE exposure, response rate and total pellets earned after the highest EtOH dose (3.5 g/kg) was lower than response rate or total pellets earned after either vehicle or 1 g/kg EtOH. There was also a significant main effect of *Age* on response rates (*F_1,18_* = 5.54, *p*<0.05) and N*umber of Pellets Earned* (F*_1,18_* = 9.29, *p*<0.01) with *post-hoc* analysis showing that, independent of CIE exposure, the animals exposed in adolescence had higher response rates and earned more pellets than the animals pre-treated in adulthood after 3.5 g/kg EtOH.

## Discussion and Conclusions

Among the primary findings in this study, we observed that adult animals were more sensitive to the initial behavioral intoxicating effect of CIE than were adolescent animals. We found that CIE exposure during adolescence altered RAM performance during an acute EtOH challenge in adulthood, whereas CIE during adulthood did not. Specifically, animals treated with CIE during adolescence made more type2 working memory errors in the RAM after acute EtOH challenge than did their age-matched controls. There was no such effect in animals exposed to CIE during adulthood. In addition, animals treated with CIE during adolescence were more active following an acute EtOH challenge in adulthood than animals treated with CIE during adulthood. Unexpectedly, we also observed that animals pre-treated with CIE as adolescents spent less time in the open portions of the RAM arms than did their age matched controls in the DNMTP task. In subsequent experiments in independent groups of rats, we found that food-motivated responding under fixed- or progressive-ratio schedules of reinforcement was not altered by CIE exposure in either age group, though there was an effect of age on operant responding. Therefore, it is unlikely that the observed differences in RAM performance in adult animals after CIE exposure during adolescence was related to changes in food-motivation.

The behavioral intoxication scores taken during CIE treatments prior to the operant task indicated that adult rats were more sensitive than adolescents to the acute effects of ethanol during the first CIE administration. This is consistent with previous studies that have reported greater sensitivity to the sedative, hypnotic, motor-impairing, anxiogenic, conditioned aversive, hypothermic and seizure-potentiating effects of ethanol in adult, compared to adolescent rats and mice [17]–[19], [21], [40], [50]–[60]. Nevertheless, there are conflicting results in the literature that may be primarily due to the route of administration, dosage and dosing regimen [61]–[63].

Our finding that acute EtOH increased type2 working memory errors in rats pre-treated with CIE during adolescence (but not in those pretreated with CIE as adults) is consistent with our earlier work [36] wherein working memory errors were increased by acute EtOH challenge more potently in rats pre-treated with CIE during adolescence than those pre-treated with CIE in adulthood. Taken together, these studies clearly indicate that CIE during adolescence renders animals more sensitive to the memory disrupting effects of acute EtOH well into adulthood, and perhaps permanently, and that the sensitivity to this long-term effect of repeated EtOH exposure is greater when the exposure occurs during adolescence than when it occurs in adulthood. Moreover, this long-term sensitivity to the acute effect of ethanol has been demonstrated on a variety of physiological, behavioral, and cognitive endpoints in previously published work [64]–[68].

That animals treated with CIE during adolescence spent less time in the open portions of the maze arms during DNMTP testing is interesting and can be interpreted in several ways. The construction of our RAM apparatus makes it physically similar to the elevated plus-maze (EPM), a test used to assess anxiety-like behavior in rodents [69]. Thus it is possible that these findings reflect an enduring effect of CIE on anxiogenesis or risk-reward integration processes. This is an interesting possibility given that the adolescent brain is less able to regulate anxiety [70], and because CIE exposure during adolescence has been shown to prolong certain adolescent neurobehavioral characteristics into adulthood, including GABAergic sensitivity and reduced motor impairment after acute EtOH challenge [34], [36], [60].

However, this interpretation must be made with due caution. The RAM was not designed or intended for the measure of anxiety-like behavior. Moreover, there are number of factors that should be taken into consideration. Discrepancies in open arm time could easily be explained by a difference in food motivation. However, consistent with Slawecki [71], we did not observe any alteration in food motivated responding under FR or PR schedules. This suggests that reward motivation is intact in CIE pre-treated animals. In addition, if this open arm time was a measure of anxiety, then similar deficits should also manifest in traditional anxiety tests such as the EPM. This was not the case after adolescent CIE exposure on anxiety-like behavior in adulthood [36]. Alternatively, these open arm results are similar to findings using a risky decision-making task [72], which highlights the complexity of risk-reward integration in rats. In this task there is evidence that the processing of the risk-reward decision making process is not dependent on reward motivation or simple EPM measures of anxiety [73] suggesting that our observation may be more complex than a simple anxiety or food motivated effect. However, much more work is needed before any conclusions can be drawn regarding the developmental effects of CIE exposure on adult risky decision making.

CIE exposure had no effect on motivation for food under baseline conditions or in response to acute EtOH challenges. In contrast, age affected food-motivated responding after an acute EtOH challenge, independent of CIE exposure. The older rats, which were exposed to either CIE or saline as adults, required more sessions to meet advancement criteria during training on FR schedules and earned fewer food rewards during the acute EtOH challenges compared to the younger rats that were exposed to either CIE or saline during adolescence. This effects was attributable to age, but not to the differences in EtOH pharmacokinetics because BECs were similar across all experimental groups. Together these findings indicate that older rats were slower to learn the operant task compared to younger rats and that older rats were more susceptible to response disruption by ethanol, but that CIE exposure had no effect learning a simple operant response, or on performance of the response after ethanol administration. In addition, the acute EtOH challenges decreased food-maintained responding in all rats independent of CIE exposure. These findings are consistent with previous findings that demonstrate reduced response rates after acute EtOH or its metabolites in ethanol-naïve rats [74], [75]. These findings also demonstrate that CIE exposure (in adolescence or adulthood) has no long-term effects on food-motivated responding. This finding suggests that the long-term changes in RAM performance produced by CIE exposure are unlikely to be due to altered sensitivity to food reward or to altered motivation to respond for food reward.

Clearly, the findings described above indicate that CIE during adolescence affects certain aspects of RAM performance in adulthood. However, the bulk of this work did not support our primary hypotheses, which focused on the interaction between age at pre-treatment (adolescent versus adult) and chronic intermittent ethanol exposure (ethanol versus saline) on traditional measures of spatial learning and working memory using the radial arm maze. For example, we found no interaction effects of Age and CIE pre-treatment on standard measures of reference memory (win-shift phase) or measures of working memory (DNMTP acquisition and random delay phases). These findings are generally consistent with other reports that show no long-term effect of adolescent CIE exposure on learning and memory [67].

Where these *Age*×*Pre-treatment* interactions were non-significant, we must weigh the possibility that the null hypothesis is true against the possibility that the experiment was under powered leading to a type II error. This invokes the age-old truth that the absence of evidence of an effect is not evidence of the absence of an effect [76]. Because it is not possible to know if the null is true, many have advocated the use of post-hoc power analyses in order to assess the probability of a type2 error [e.g., Onwuegbuzie and Leech [77]]. However, there is a growing literature documenting the misuse and misinterpretation of post-hoc power analyses. Briefly, there is a strong inverse relationship between power and p (type I error probability) for a given effect size, due in large part to the fact that both (power and p) are dependent on sample size. Therefore, any statistically non-significant effect will have inadequate power [see Hoenig and Heisey [78] for review]. As a result, many have turned to a procedure known as equivalency testing (ET).

Equivalency testing is increasingly common in the clinical trials literature where there is an effort to demonstrate that a new drug is equivalent to an older drug [45]. In other words, there is an effort to demonstrate that the null hypothesis is true (no difference between drugs) using mean difference effects sizes such as Cohen's d or Hedge's G. Unfortunately, this type of effects size represents group differences (e.g., drug A versus drug B) on a dependent variable (e.g., level of depression) in standard deviation units. Therefore, it is generally not useful in ANOVA models where there are more than two levels of a particular independent variable or where the interaction of two independent variables is the effect of interest. For example, in the present study, our primary interest is in the interaction between the *Age* at *Pre-treatment* (adolescent versus adult) and the type of *Pre-treatment* (chronic intermittent ethanol versus saline). In this model, there is no simple group A versus group B comparison. Because one does not make simple pairwise comparisons in ANOVA models, effect size is most often expressed as the proportion of variance in the dependent variable accounted for by the independent variable. In light of this, ET is not strictly applicable. However, the essential logic remains relevant.

As in traditional ET, we selected a minimum effect size criterion (0.05), against which we compared the observed effect size (η_p_^2^+90% C.I.; see Methods above). Where η_p_^2^ (+90% C.I.) falls below the criterion, the effect is to be considered functionally irrelevant. That is, if we are 90% confident that the *Age*×*Pre-treatment* interaction accounts for less than 5% of the variance in a particular dependent variable, then the null hypothesis should be considered true - for all practical purposes. Where η_p_^2^ (+90% C.I.) overlaps or exceeds the criterion, but otherwise remains statistically non-significant, the effect is to be considered of some potential functional relevance and worthy of further investigation. It is worth emphasizing that the sole purpose of this strategy is to differentiate those findings that are unlikely to bare useful outcomes in future research from those that may be worthy of further investigation. The latter of which should not be considered statistically significant.

This η_p_^2^ criterion (0.05 in the present study) should not be confused with the alpha described in the Methods section above. The alpha criterion is used to identify those effects that are statistically significant. These constructs are unrelated. The η_p_^2^ criterion is an upper limit for the effect size (η_p_^2^+90% CI) to differentiate those effects that might be considered meaningful versus those that should not be considered meaningful. This η_p_^2^ criterion was chosen a priori based on several observations from the human binge drinking and related literature. Theoretically, binge drinking and its long-term cognitive sequelae are 100% preventable and avoidable. Any controllable behavior having a presumably adverse effect on cognition would seem worthy of careful investigation. Moreover, cognition is integral to one's ability to function in modern society. As a result, we argue that any variable that might account for more than 5% of the variability in cognition is worthy of further investigation. Finally, our ability to assess cognition in rodents is less sophisticated than our ability to assess cognition in humans. Therefore, the proportion of variability attributed to an independent variable in a rodent model may scale up to a larger proportion of variability in a human model.

Putting this criterion (η_p_^2^ = 0.05) into another perspective underscores its conservative nature. In designing an animal behavior experiment with an expected effect size equal to our criterion (η_p_^2^ = 0.05), we can calculate the required sample size to achieve power = 0.8 (assuming alpha = 0.05) for the interaction term in a 2×2 ANOVA design (similar to the design used in the present study). With these assumptions (η_p_^2^ = 0.05, power = 0.8, alpha = 0.05), the experiment would need to be designed with 38 animals per cell (152 animals total) to achieve the desired power [79]. Many would carefully reconsider their design or their endpoints before embarking on a study where so many animals would be needed to achieve the minimum adequate power.

**Figure 9:**
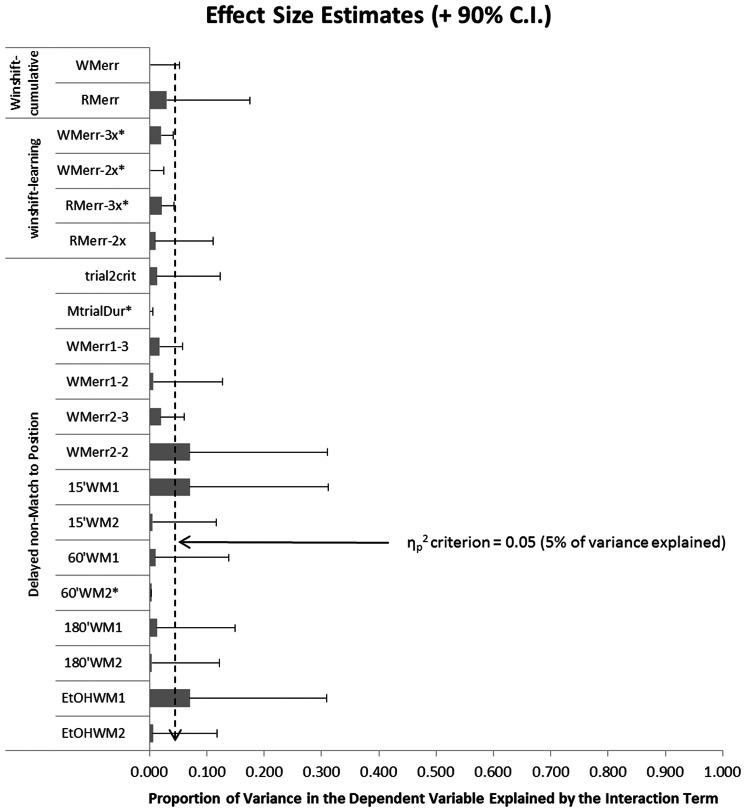
Effect sizes and 90% confidence intervals for non-significant Age x Pre-Treatment interaction effects relative to the a priori 0.05 criterion. Observed effect sizes (+90% C.I.) falling below the criterion are considered functionally irrelevant (marked as *). Effect sizes (+90% C.I.) exceeding or overlapping the criterion, but otherwise remaining non-significant, may be consider to be of potential interest in future research. Dependent variables listed include: WINSHIFT cumulative working (WMerr) and reference (RMerr) errors; WINSHIFT working (WMerr-3x) and reference (RMerr-3x) memory errors for the Age x Pre-Treatment x Day interaction, and working (WMerr-2x) and reference (RMerr-2x) memory errors for the Age x Pre-Treatment interaction collapsing across days; DNMTP trials to criterion (trial2crit), mean trial duration (MtrialDur), type1 (WMerr1–3) and type2 (WMerr2–3) working memory errors for the Age x Pre-Treatment x Day interaction, and type1 (WMerr1-2) and type2 (WMerr2–2) working memory errors for the Age x Pre-Treatment x interaction collapsing across days; cumulative type1 (15′WM1) and type2 (15′WM2) working memory errors during the 15 m delay; cumulative type1 (60′WM1) and type2 (60′WM2) working memory errors during the 60 m delay; cumulative type1 (180′WM1) and type2 (180′WM2) working memory errors during the 180 m delay; cumulative type1 (360′WM1) and type2 (360′WM2) working memory errors during the 360 m delay; and, cumulative type1 (EtOHWM1) and type2 (EtOHWM2) working memory errors following the EtOH challenge.

During the course of the RAM portion of these experiments, the Age x CIE pre-treatment interaction effect was observed to be non-significant on 20 separate dependent variables. The effect sizes (+90% C.I.) corresponding to five of these dependent variables were fully below our 0.05 criterion (see variables marked* in Figure 9). It was also observed that the *Age*×*Pre-treatment* interaction never accounted for more than 7% of the variance in any of these 20 dependent variables (just above our 0.05 criterion). By the rationale provided above, these remaining variables might be considered fruitful avenues for further research. However, these remaining effect sizes are sufficiently small that further investigation of the *Age*×*Pre-treatment* interaction on the corresponding variables should only be under taken in the presence of a moderating variable. That is, we believe that the delayed effect of adolescent CIE exposure on learning and memory in adulthood may only be meaningful in the presence of a neurobiological stressor in adulthood (e.g., social stress, pharmacological challenge, or neuropathology).

It is also important to note that these negative results should not be taken as an indication that binge drinking is safe. It may well turn out that there are few or only mild long-term consequences of binge drinking on cognition or behavior. However, much more research needs to be done both at the pre-clinical and clinical level before any such conclusion would be justified. More importantly, the absence of long-term effects of binge drinking does not mean that binge drinking in adolescence is without consequence. Indeed, the proximal effects of binge drinking among adolescents and young adults are now well established in the human literature. Such effects include poorer academic performance, unprotected and unwanted sex, driving under the influence, and physical aggression and violence, to name just a few [80], [81]. Binge drinking is a dangerous and maladaptive pattern of alcohol consumption and should be avoided at any age.

The growing awareness that adolescence represents a period of selective vulnerability to the enduring effects of repeated EtOH exposure is supported by the observation that none of the long-term effects reported above were observed after CIE exposure in adulthood. Moreover, these effects are not explained by differences in food motivated responding. Taken together, these data suggest that the long-term effects of adolescent CIE exposure are subtle and are not manifest in simplistic behavioral paradigms. As such, more complex behavioral approaches in conjunction with pharmacological or physiological challenges may be required to elucidate the underlying cognitive deficits observed following adolescent CIE exposure. It should also be noted that the CIE regimen used here was only a moderate EtOH exposure. The use of higher and/or more frequent dosing may extend the present findings. Future work is needed to determine the extent to which CIE exposure in adolescents (versus CIE in adulthood) may affect inhibitory behavioral control in the context of a spatial learning paradigm. Further work is also necessary to determine whether CIE exposure during adolescence has adverse effects on risk-reward integration during adulthood. Deficits in risk-reward integration may be particularly relevant in the later development or maintenance of alcohol use disorders.
